# Supplementary material for: Structure and stabilization of the antigenic glycoprotein building blocks of the New World mammarenavirus spike complex
Source: mBio. 2025 Jun 13;16(7):e01076-25. doi: 10.1128/mbio.01076-25 (PMC12239557; doi:10.1128/mbio.01076-25)
Supplement: Supplemental material — Supplemental figures and table. [file mbio.01076-25-s0001.pdf]

Supplementary Figures and Tables for:

Structure and stabilization of the antigenic glycoprotein building blocks of New World mammarenavirus spike complexes

Guido C. Paesen, Weng M. Ng, Simon Kimuda, Geoff Sutton, Katie J. Doores,

Thomas A. Bowden

This document includes:

Figs. S1 to S7
Table S1

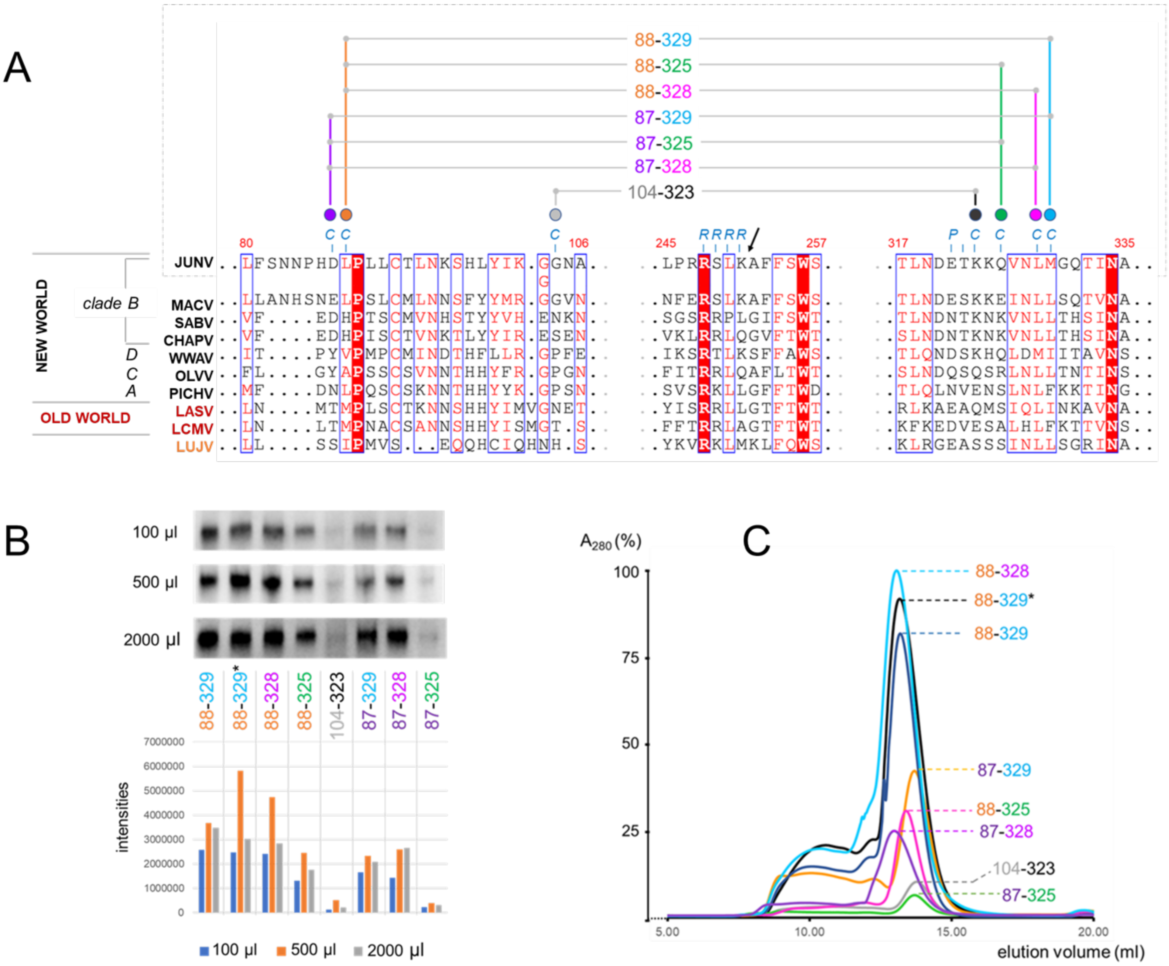

Fig. S1. Alignment of JUNV GP1-GP2 (top sequence) with heterodimers of other NW- and OW-arenaviruses, and the OW-outlier Lujo virus (LUJV). A list of the abbreviations of virus names used is provided in the Material and Methods of the main text. Only sequence fragments relevant to the design of the JUNV constructs are shown. In the JUNV GP1–GP2^{e} constructs, a furin cleavage site (RRRR; blue letters) replaced the native SKI-1 site (black arrow), and Glu321 was mutated into a proline (P), except for a version of GP1$^{88\text{-}329}$GP2^{e} (suffixed with an asterisk in panels *B* and *C*). Cysteines (C; blue) introduced to allow inter-chain disulfide-bond formation are annotated with color-coded spheres. The GP1–GP2^{e} constructs of other arenaviruses tested in this study, including MACV, have cysteines introduced at positions equivalent to those in the JUNV GP1$^{88\text{-}329}$GP2^{e} construct. (**B**) Western blots of expressed JUNV GP1–GP2^{e} constructs, named after their inter-chain disulfide bond. For each construct, 20-mL cell cultures were infected with 100, 500, and 2,000 µL of baculovirus stocks. After 72h, protein from 150 µL of the culture medium was bound onto StrataClean resin (Agilent) and subjected to non-reducing SDS-PAGE and western blotting. (**C**) SEC profiles of JUNV GP1–GP2^{e}s from 2-L Sf9 cultures following purification over streptactin-XT resin. The absorbance at 280 nm of the best performing construct was set to 100%. Whilst expression levels of a given construct differed from one experiment to another, the yields obtained with the GP1$^{88\text{-}329}$GP2^{e} and GP1$^{88\text{-}328}$GP2^{e} constructs were superior to those obtained with GP1$^{87\text{-}329}$GP2^{e}, GP1$^{87\text{-}328}$GP2^{e} and GP1$^{88\text{-}325}$GP2^{e}, whilst expression of GP1$^{87\text{-}325}$GP2^{e} and GP1$^{104\text{-}323}$GP2^{e} was relatively low.

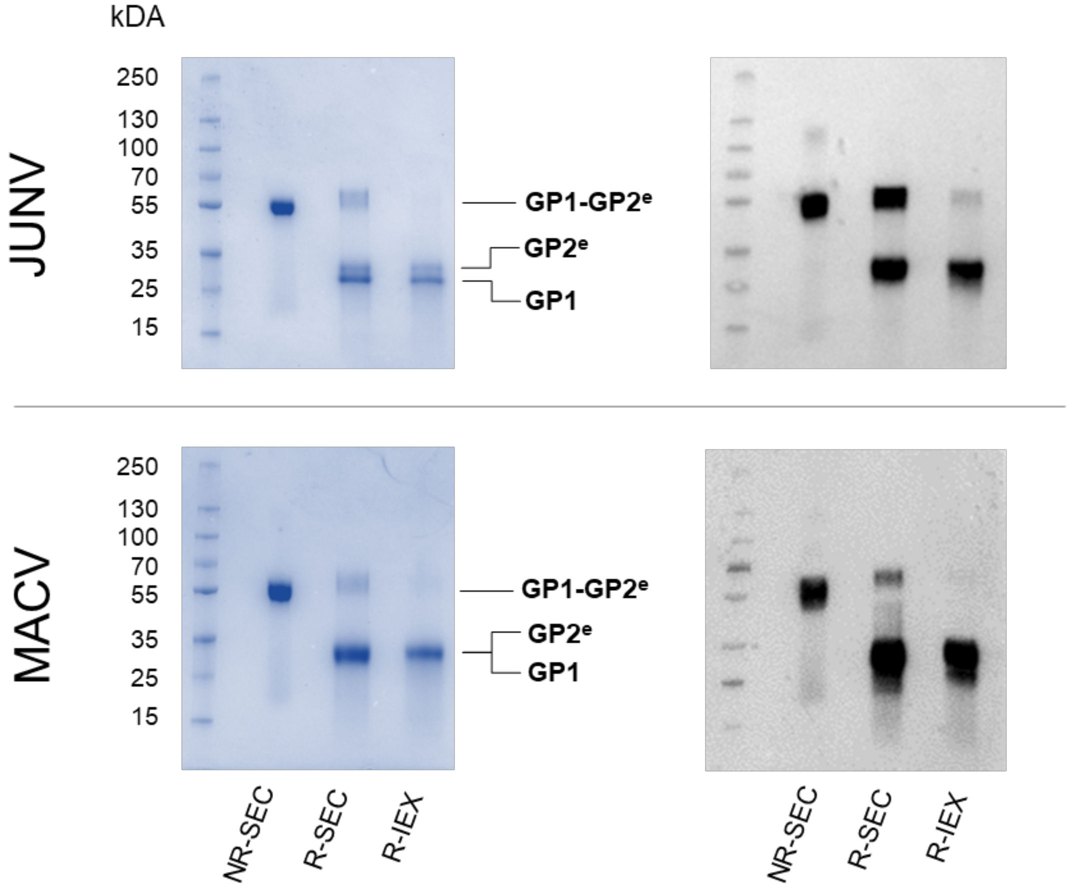

Fig. S2. SDS-gels (left) and western blots (right) of purified JUNV GP1$^{88\text{-}329}$GP2^{e} and MACV GP1$^{88\text{-}340}$GP2^{e} protein. Lane NR-SEC shows SEC-purified, non-reduced protein. Running close to the 55 kDa protein marker, it corresponds to the disulfide-bonded heterodimer. Consistently, the reduced form of the protein (lane R-SEC) runs as two ~30 kDa bands, corresponding to the GP1 and GP2^{e} subunits, with non-glycosylated forms having calculated molecular masses of ~22.4 and ~22.9 kDa, respectively. In MACV, where GP1 has an additional glycosylation sequon and is 11 residues longer than in JUNV, the GP1 and GP2 bands overlap. The antibody used in the western blots recognizes the C-terminal twin-strep tag, showing the non-reduced heterodimer and the GP2^{e} subunit, but not the GP1 subunit. After SEC, a feint ~55 kDa band is observed, which likely corresponds to protein that did not undergo furin cleavage. Most cleaved protein is removed during ion-exchange chromatography (lane R-IEX).

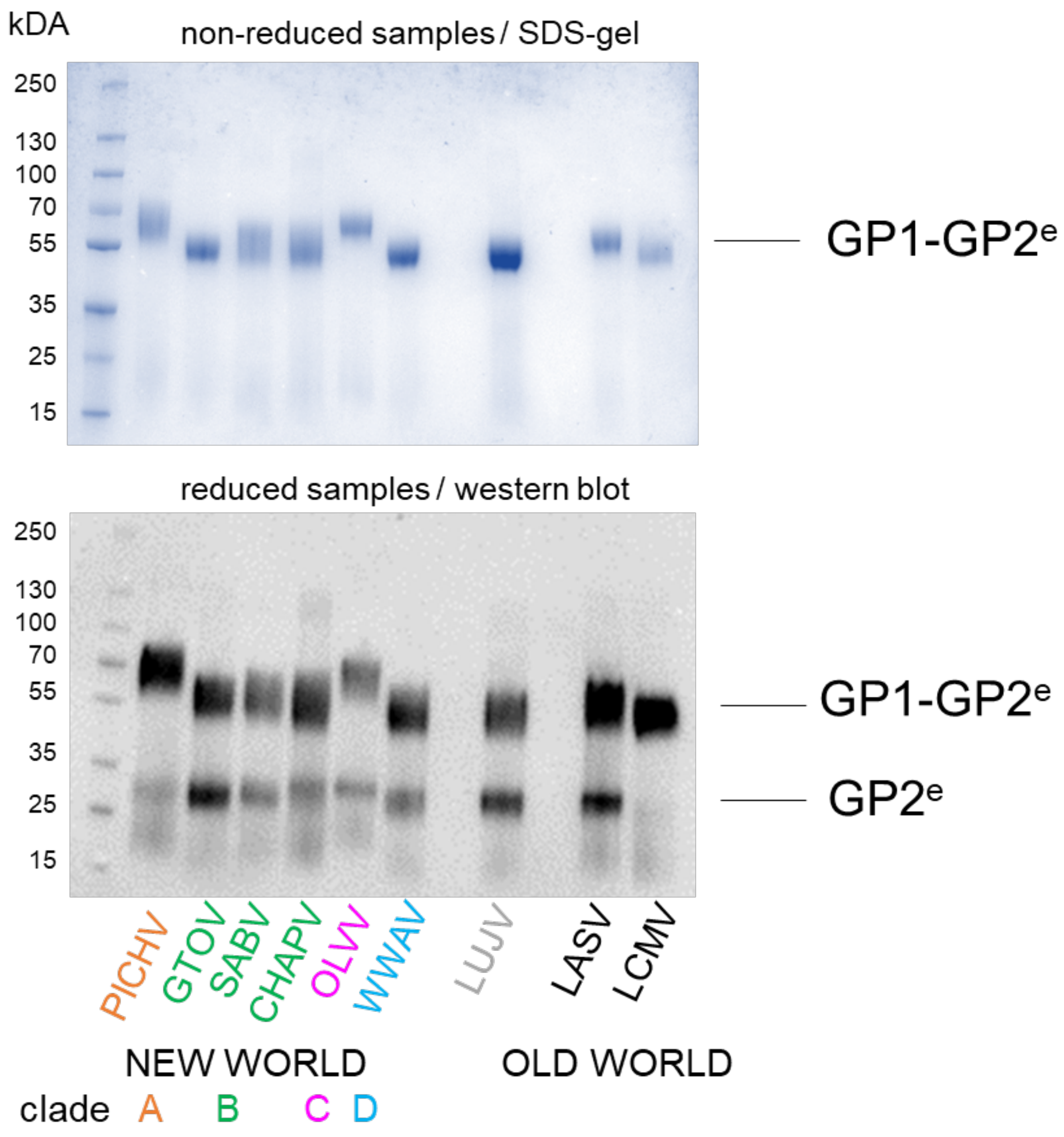

Fig. S3. SDS-gel (top) and western blot (bottom) of mammarenavirus GP1–GP2^{e} proteins. Non-reducing SDS-gel (top) and western blot of reduced samples (bottom), showing GP1-GP2^{e} proteins of representative NW- and OW- mammarenaviruses stabilized with an inter-chain disulfide bond whose position was based on that in JUNV GP1$^{88\text{-}329}$GP2^{e}. The SKI-1 cleavage site was replaced with a furin site in all constructs and a proline was introduced by site-directed mutagenesis at the position of the E321P mutation in the JUNV construct. The non-reduced proteins run at about 55 kDa. The absence of ~25 kDa bands corresponding to free GP1 and GP2^{e} subunits under non-reducing conditions indicates that the GP1–GP2 complex is covalently held together by a disulfide bond, or that the GP1GP2 chain did not undergo furin cleavage, or both. The western blot, which detects the C-terminal twin-strep tag in the reduced samples, shows uncleaved protein and free GP2^{e} subunits, which must have arisen from furin-cleaved, disulfide-linked GP1–GP2^{e} complexes. Taken together, the SDS-gel and western blot indicate that the disulfide bond is formed, although furin cleavage is likely incomplete. The expression yields of the NW proteins were comparable to those of the JUNV and MACV constructs, and those of the LASV, LCMV, and LUJV proteins were lower.

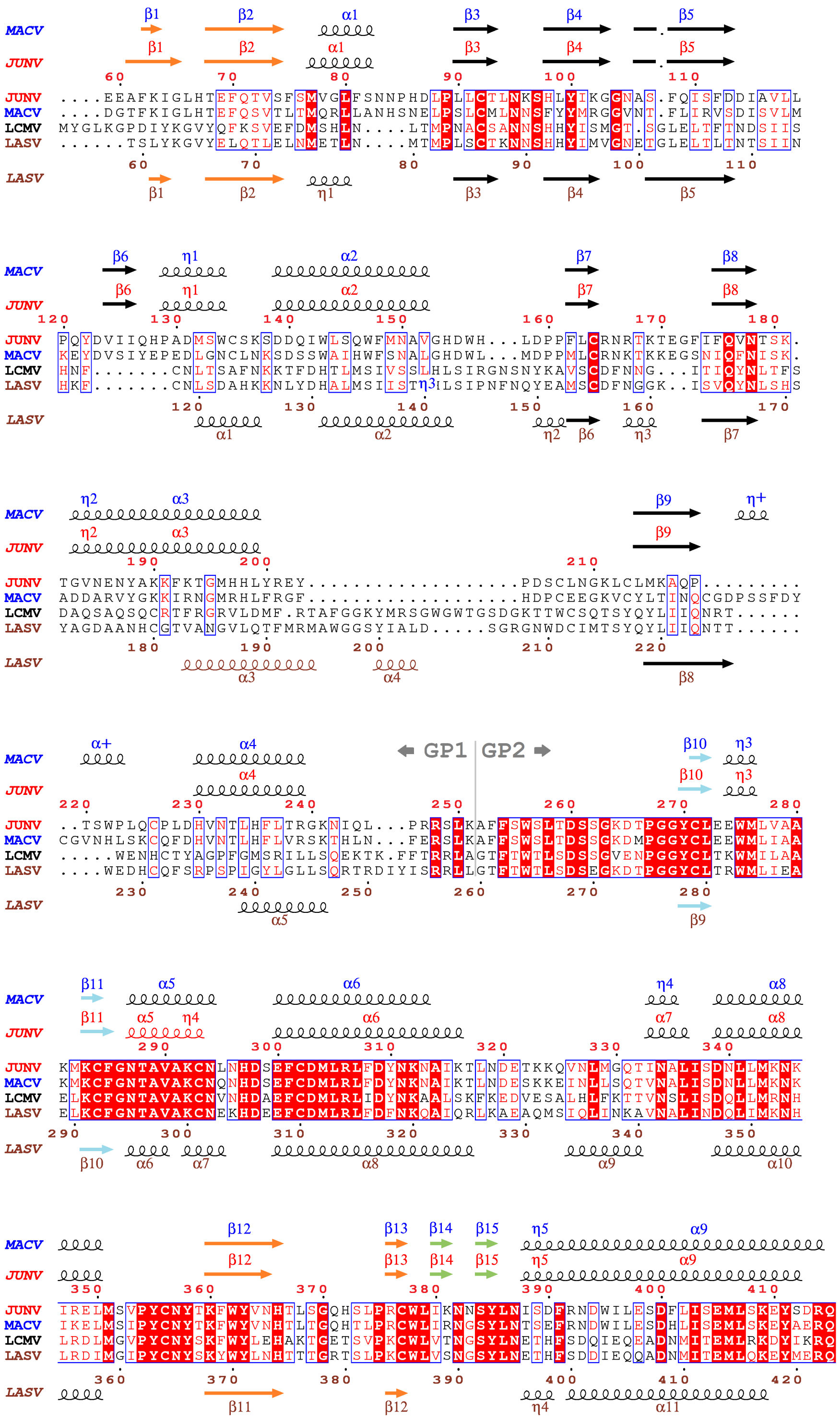

Fig. S4. Structural alignment of GP1–GP2[e] sequences of JUNV, MACV, LCMV and LASV. Secondary-structure elements observed in JUNV GP1–GP2 and MACV GP1–GP2 crystal structures are shown above and below the alignment (LASV; PDB code 5VK2, chain C). The symbols α, β and η indicate α-helices, β-strands and 3_{10}-helices, respectively. The arrows denoting the β-strands are colored according to the sheet they belong to: orange for the sheet formed by the GP1 latch region and the GP2 T-loop region, black for the central β-sheet in GP1, light blue and green for small, 2-strand β-sheets in GP2. The secondary structure elements in the MACV GP resemble those in JUNV, except that a 3_{10} helix (η4) replaces α-helix 7. Moreover, the extra, 11-residue peptide in MACV (AA 220-230) contains a 3_{10} and an α-helix (termed η+ and α+ in the alignment).

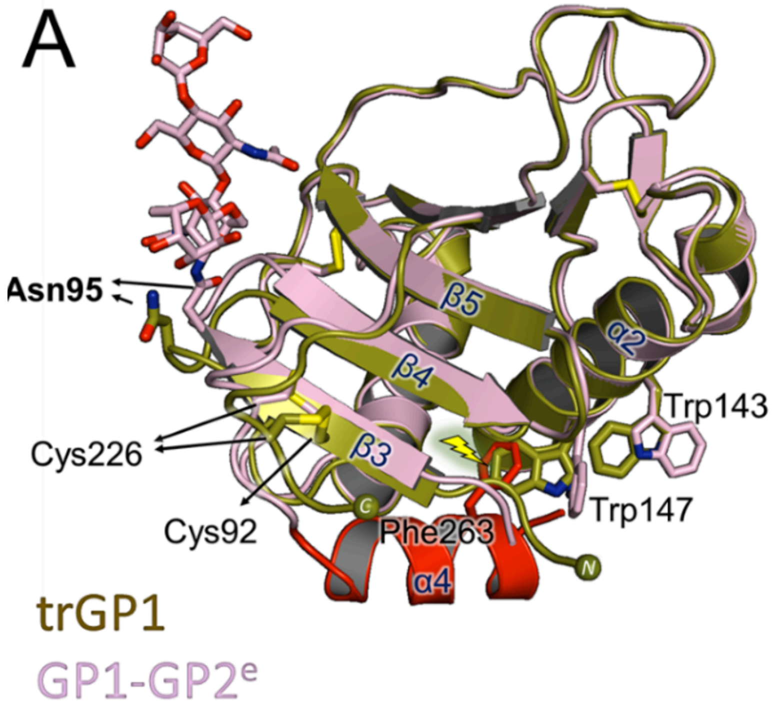

Fig. S5. Comparison of GP2-bound to GP2-free GP1. Superposition of JUNV trGP1 (*i.e.* the truncated GP1 construct lacking the N-terminal strap and C-terminal α4 helix; PDB 7QU2; olive-colored cartoon) onto the (strap-less) GP1-subunit of $GP1^{88\text{-}329}GP2^{e}$ (pink, except for α4; red). Asn95-linked glycan and side groups of relevant residues are shown as sticks. The N- and C-termini of the trGP1 structure are indicated by spheres. The structures differ in the trajectory and size of their β3-β4 loops, and in the position of Asn95, which appears glycosylated in the heterodimer structure. These differences likely relate to the absence of the α-helix 4 in the trGP1s, which, in the GP1–$GP2^{e}$ structures, stabilizes the β3 and β4 strands. Specifically, α4 may guide the course of the nearby (β9-α4) loop, which is linked to β3 via a disulfide bond (Cys92–Cys226). Additionally, α4 forms hydrophobic interactions with β4, as observed in Fig. 6 of the main article. Unrelatedly, Phe236 on α4 also sterically restricts the orientation of the Trp147 side chain, which in turn limits that of Trp143. Although the side chains of these residues appear flexible in the heterodimer, as indicated in the crystal, they cannot assume the orientations observed in the trGP1s, as this would result in clashes between the Trp147 and Phe236 side-groups (yellow thunderbolt).

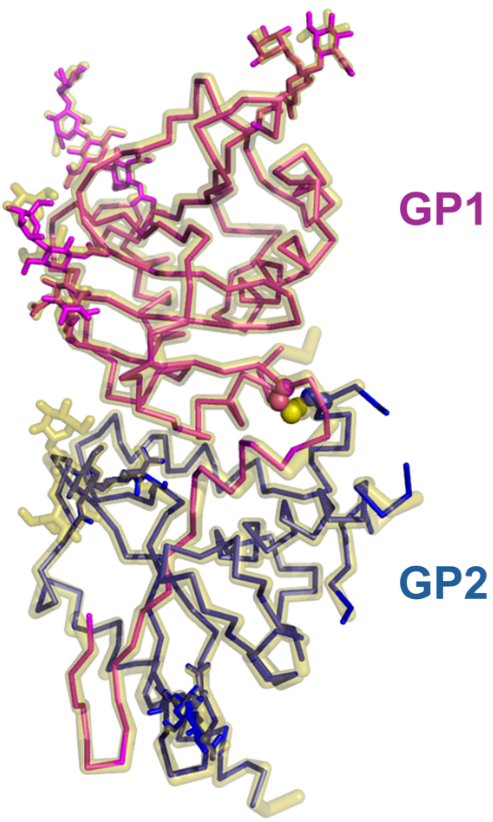

Fig. S6. Superposition of the JUNV GP1$^{88\text{-}329}$GP2^{e} and JUNV GP1$^{88\text{-}329}$GP2^{e*}structures. The asterix in JUNV GP1$^{88\text{-}329}$GP2^{e*} indicates that this construct lacks the extra E321P mutation. Structures are shown in ribbon presentation. The yellow, wider ribbon corresponds to JUNV GP1$^{88\text{-}329}$GP2^{e*}, the narrower magenta (GP1) and blue (GP2^{e}) ribbons represent JUNV GP1$^{88\text{-}329}$GP2^{e}. The interchain Cys88–Cys329 disulphide bridge is shown as spheres. Glycans are shown as sticks and are colored according to the chain they are attached to. The RMSD between the two structures is ~0.4 Å across aligned Cα pairs.

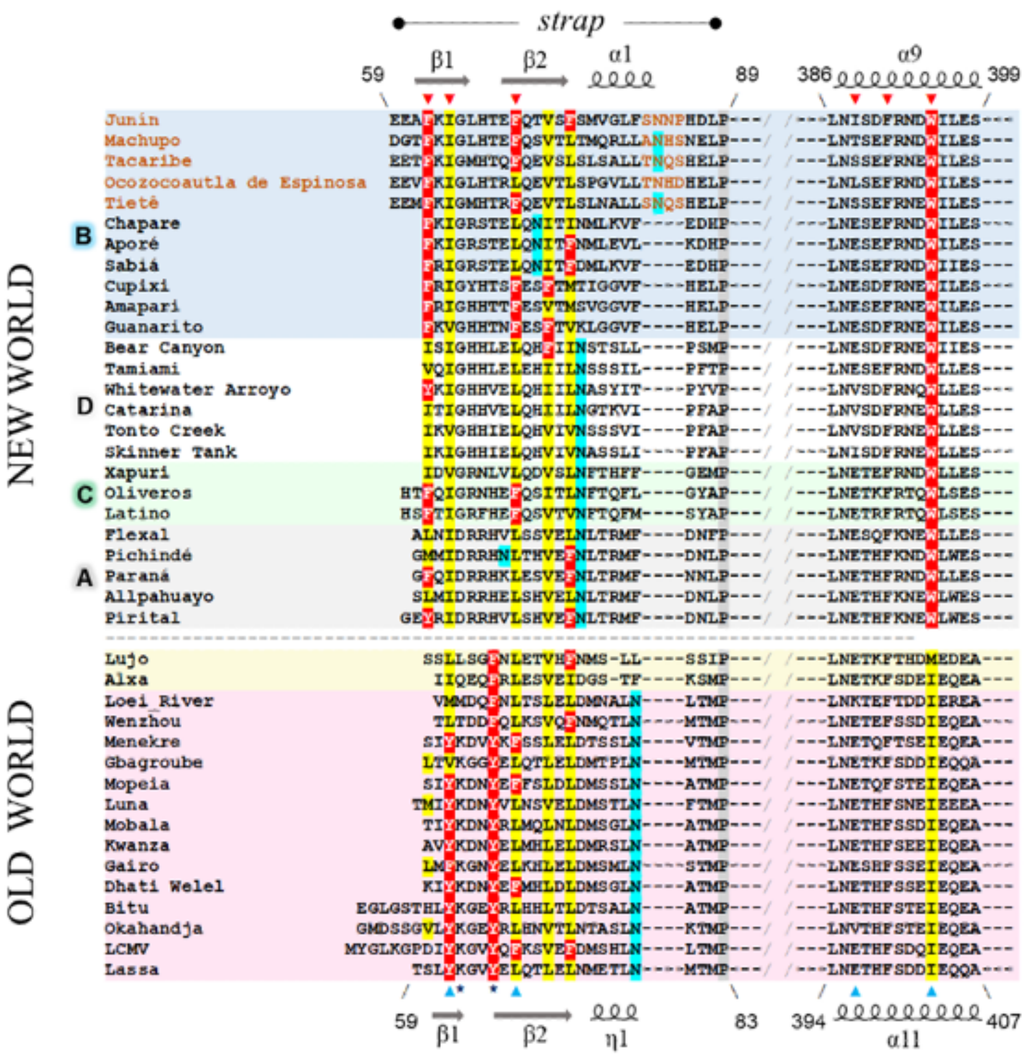

Fig. S7. Alignment showing conserved hydrophobic residues in the strap region and part of the C-terminal α-helix of GP2[e]. Sequences of NW viruses are shown against a blue (clade B), white (D), green (C) or grey (A) background, while those of OW viruses and OW-outliers against a pink and yellow background, respectively. The clade B viruses labelled in orange represent a subset in which the α1-β3 loop of the strap is extended with 4 amino acids (also colored orange). In some of these viruses, the extension contains an N-glycosylation sequon (with the asparagine highlighted in blue). In the MACV GP1–GP2 crystal structure, the Asn is glycosylated. This sequon structurally aligns with that conserved at the C-terminus of η1-helix in the OW viruses, which is glycosylated in the LASV and LCMV structures. Residue positions in the JUNV and LASV sequences are shown above and below the alignment, respectively. Conserved hydrophobic residues in the strap are highlighted in red (aromatic residues) and yellow (non-aromatic). In the C-terminal helix, only Trp395 in NW viruses and its Ile405 counterpart in OW viruses are conserved (yellow). Arrowheads indicate residues involved in the strap-GP2 interactions in JUNV (above the alignment, in red), and in LASV (below the alignment; blue). Asterisks denote strap residues interacting with the SSP.

Table S1. Crystallographic data collection and refinement statistics.

**Data Collection**		**JUNV GP1$^{88-329}$GP2^{e}-JUN1**	**MACV GP1$^{88-340}$GP2^{e}-MAC1**	**JUNV GP1$^{88-329}$GP2^{e}*-JUN1**
Beamline		DLS I03	DLS I04-1	DLS I24
Wavelength (Å)		0.97625	0.91788	0.9999
Space Group		*C* 1 2 1	*P* 1 21 1	*C* 1 2 1
Cell Dimensions	*a, b, c* (Å)	225.8, 72.0, 80.2	46.7, 73.6, 185.0	226.5, 73.0, 80.7
	α, β, γ (°)	90, 95.9, 90	90, 91, 90	90, 95.8, 90
Resolution range (Å)		112.30-2.09 [2.13-2.09]	184.93-2.41 [2.45-2.41]	112.66-2.55 [2.59-2.55]
Rmerge		0.202 [2.604]	0.272 [2.531]	0.158 [2.287]
I/σ (I)		6.55 [0.36]	6.5 [0.5]	7.2 [0.8]
$CC_{1/2}$		0.993 [0.318]	0.989 [0.314]	1 [0.4]
Completeness (%)		100 [99.95]	99.9 [98.4]	99.9 [100]
Multiplicity		7.0 [6.6]	7.0 [7.3]	4.0 [4.1]

Refinement

Resolution (Å)		79.81-2.09	47.25-2.41	80.32-2.55
No. reflections		75,109	48,642	42,768
R_{work}/R_{free}		0.213/0.248	0.230/0.269	0.202/0.237
No. atoms	Protein	5,982	6,074	6,005
	Ligand	203	233	226
	Water	178	115	175
Average B-factor	Protein	57.5	65.8	77.4
	Ligand	81.5	98.4	102.5
	Water	47.5	51.2	68.3
Ramachandran (%)	Favored	97.5	95.9	96.1
	Allowed	2.4	4.1	3.9
	Outlier	0.1	0	0
RMSD	Bond lengths (Å)	0.002	0.002	0.003
	Bond angles (°)	0.57	0.54	0.60

Numbers between brackets represent values for the highest-resolution shells.
JUNV GP1$^{88-329}$GP2^{e}* refers to the JUNV GP1$^{88-329}$GP2^{e} construct in which E321 was not mutated into a proline.
